# Enterprise characteristics and occupational health literacy among essential service workers in Guangdong Province, China: a cross-sectional study

**DOI:** 10.3389/fpubh.2025.1632185

**Published:** 2025-08-26

**Authors:** Shanyu Zhou, Huiqing Chen, Junle Wu, Bing Xia, Xinyang Yu, Manqi Huang, Min Yang

**Affiliations:** Guangdong Province Hospital for Occupational Disease Prevention and Treatment, Guangzhou, Guangdong, China

**Keywords:** occupational health literacy, essential service workers, enterprise characteristics, cross-sectional study, tertiary industry

## Abstract

**Background:**

Essential service workers are exposed to significant occupational health risks. Low occupational health literacy (OHL) has been linked to increased risks of occupational injuries and work-related illnesses. However, limited evidence exists regarding the OHL of essential service workers and the impact of enterprise characteristics on OHL. This study aimed to examine the associations between enterprise characteristics and OHL among essential service workers in Guangdong Province, China.

**Study design:**

Cross-sectional study.

**Methods:**

A cross-sectional survey was conducted among 2,640 essential service workers from the environmental sanitation (*n* = 880), transportation (*n* = 880), and express and food delivery industries (*n* = 880), using multi-stage cluster random sampling. OHL was assessed using the National Occupational Health Literacy Monitoring Questionnaire, which measures four dimensions: knowledge of occupational health laws, knowledge of occupational health protection, skills of occupational health protection, and healthy work patterns and behaviors. Multivariable logistic regression was used to examine associations between enterprise characteristics (scale, ownership, industry) and OHL, with stratified analyses by monthly income.

**Results:**

The overall OHL level among essential service workers was 52.8%. Working in small and micro enterprises (adjusted OR = 1.42, 95% CI: 1.17–1.72) and private enterprises (adjusted OR = 1.28, 95% CI: 1.05–1.55) was associated with higher OHL, while employment in the express and food delivery industry was associated with lower OHL (adjusted OR = 0.64, 95% CI: 0.49–0.82). Monthly income significantly moderated these associations, with stronger effects observed in higher-income groups.

**Conclusion:**

Enterprise characteristics significantly influence OHL among essential service workers. Targeted OHL promotion strategies should be developed based on enterprise characteristics, with particular attention to large, state-owned enterprises and express and food delivery workers.

## Introduction

Occupational health literacy (OHL) refers to workers’ consciousness and capacity to access fundamental occupational health knowledge, implement healthy work and lifestyle practices, mitigate risks of occupational diseases and work-related illnesses, and maintain and promote their personal health status (1). The World Health Organization recognizes health literacy as a critical determinant of health (2). In the workplace, adequate OHL empowers workers to make health-appropriate decisions, comply with safety protocols and actively participate in health promotion activities (3, 4). Conversely, research has demonstrated that low OHL levels are associated with increased occupational injuries, reduced work ability, and a higher prevalence of work-related illnesses (5–8), highlighting the need to address OHL as a public health priority. Moreover, OHL can be improved through interpersonal support and organizational initiatives that promote healthy behaviors and take a comprehensive approach to employee health (9, 10).

Despite the recognized importance of OHL for worker health and safety, existing research has largely concentrated on traditional industries, with less attention paid to occupational groups that are essential to urban functioning. In China, essential service workers—including environmental sanitation workers, transportation drivers, and express and food delivery personnel—not only constitute a significant proportion of the workforce, but also play a fundamental role in maintaining urban operations (11). These workers are routinely exposed to various occupational hazards, including adverse weather conditions, irregular work hours, high physical demands, traffic accidents, and psychosocial stress (12). However, despite their occupational vulnerability, limited surveillance data exist regarding their OHL levels and associated factors. China has experienced a significant structural transformation in its labor force, with a marked shift toward the tertiary sector. As China’s largest employment hub, Guangdong Province encompasses approximately one-tenth of the nation’s workforce. By 2021, the tertiary industry employed 37.54 million workers, accounting for 53.1% of the province’s total workforce—a 4.5% increase from 2017 (13, 14). This transformation is particularly manifested in the rapid growth of platform-based essential services, where workers face unique occupational health challenges.

Research suggests that enhancing health literacy requires consideration of both individual factors and organizational influences. In the workplace context, this interplay between individual and organizational factors becomes especially critical for OHL (15). Given the unique occupational context of essential service workers, it is important to further explore the determinants that may influence their OHL, particularly those related to the organizational and structural environment in which they work. Among these, enterprise characteristics—such as enterprise scale, ownership, and industry—may play a significant role by shaping organizational health policies, access to occupational health services, training opportunities, and workplace health culture (16–18). However, empirical evidence examining the relationship between enterprise characteristics and OHL remains limited. Existing studies have primarily focused on traditional manufacturing sectors, while essential service industries have received insufficient attention (19). Understanding how enterprise characteristics affect OHL is crucial for developing targeted interventions and improving occupational health protection systems.

Therefore, this study aimed to assess OHL levels among essential service workers in Guangdong, analyze the associations between enterprise characteristics and OHL, and explore the potential moderating effect of workers’ income levels in these relationships. The findings will contribute to the evidence base for the development of targeted occupational health interventions in the essential service sector.

## Methods

### Study design and sampling

This cross-sectional study was conducted as part of the Guangdong Province Key Population Occupational Health Literacy Surveillance Project in 2022. According to the project protocol, each selected district required a minimum of 220 participants from each industry. With four districts and three industries involved, this determined the total target sample size of 2,640 participants.

A multistage stratified random sampling method was employed. Initially, four cities were randomly selected from Guangdong Province, followed by the selection of one district within each city: Baiyun district in Guangzhou, Xiangzhou district in Zhuhai, Dianbai district in Maoming, and the municipal district of Dongguan. Within each selected district, enterprises from three targeted industries—environmental sanitation, transportation, and express and food delivery—were randomly sampled. Specifically, 1–3 sanitation institutions, 2–3 transportation companies (including taxi, public transportation, freight, and ride-hailing platform companies), and 1–3 express or food delivery companies were selected in each district. Eligible sanitation workers, drivers, couriers, and food delivery workers from these enterprises who voluntarily agreed to participate were recruited for the survey.

**Figure 1:**
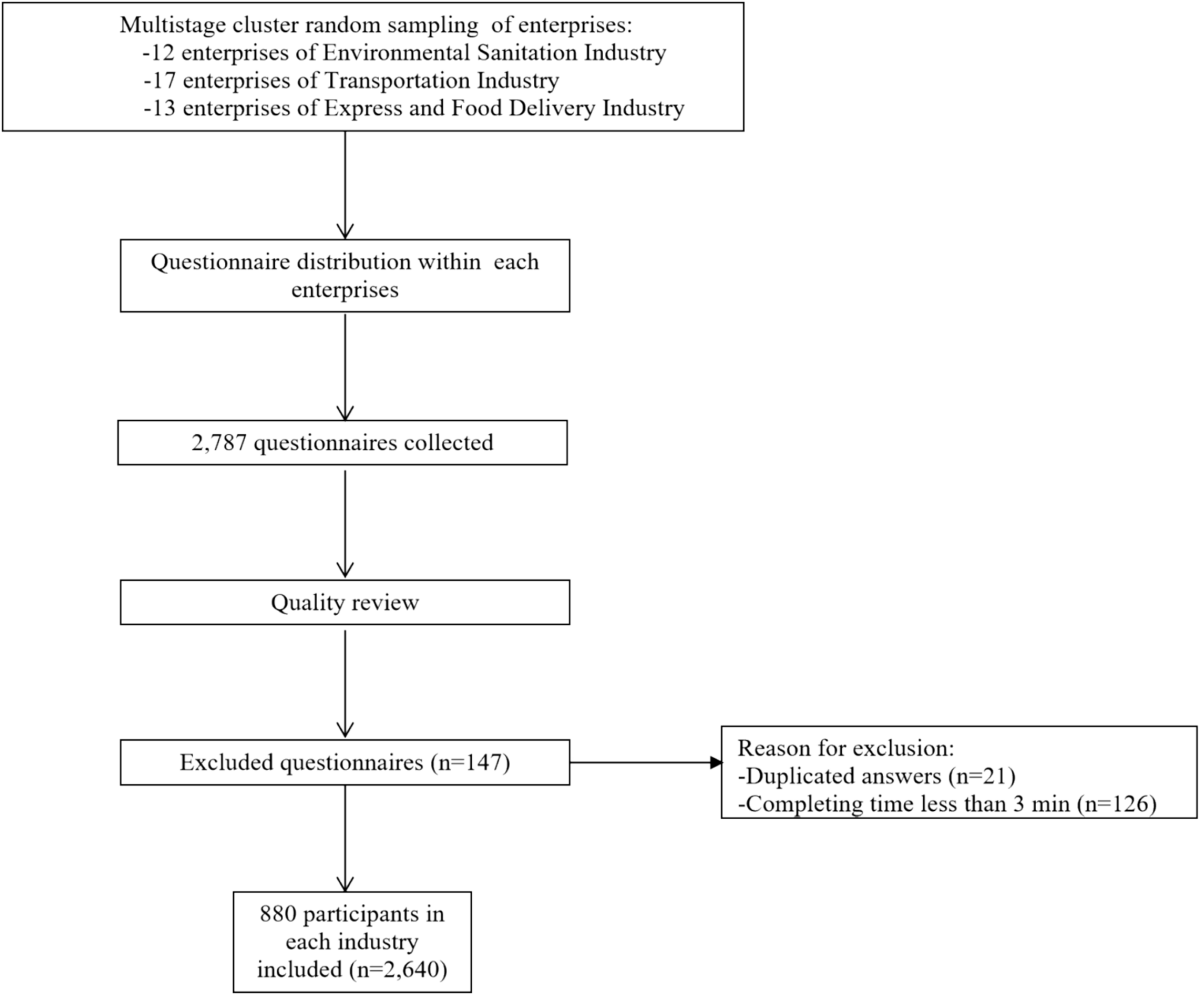
Flow chart of sample selection.

A total of 42 enterprises were selected: 12 from the environmental sanitation industry, 17 from the transportation industry, and 13 from the express and food delivery industry. The questionnaires underwent real-time review, and data collection stopped automatically when 220 valid responses were reached for each industry in each district. A total of 2,787 questionnaires were collected. After quality review, 147 questionnaires were excluded (21 due to duplicated answers and 126 due to completion time less than 3 min), resulting in 2,640 valid questionnaires, with 880 individuals from each industry (Figure 1).

### Questionnaire and data collection

The OHL was assessed using the National Occupational Health Literacy Monitoring Questionnaire, which was developed by the National Institute for Occupational Health and Poison Control and validated in previous studies (20). The questionnaire consisted of two sections: (1) basic demographic and occupational information, including sex, age, ethnic group, marital status, household registration status (hukou type), enterprise scale, ownership, industry, educational level, monthly income, working years and so on. (2) An OHL assessment with 41 items across four dimensions: knowledge of occupational health laws (11 items), knowledge of occupational health protection (14 items), skills of occupational health protection (4 items), and healthy work patterns and behaviors (12 items). The questionnaire was administered through the National Occupational Health Literacy Monitoring Survey Information System^1^. To ensure data quality and prevent potential online data collection bias, the system generated unique QR codes for questionnaire access, preventing duplicate submissions (21). Participants completed the survey anonymously by scanning these QR codes with smartphones under supervised conditions. A comprehensive data quality assurance mechanism was incorporated, including mandatory response fields to ensure data completeness, automated logical consistency checks, and backend data verification with real-time completion status tracking. Trained research staff conducted on-site supervision during questionnaire completion, providing clarification on items when necessary while avoiding response influence.

### Measures

#### Dependent variables

According to the ‘Standards for Calculating Occupational Health Literacy Levels among Key Populations (2022), both overall and dimension-specific OHL levels were assessed (22). The scoring system assigned different weights to questionnaire items: in the Basic Skills of Occupational Health Protection dimension, one multiple-choice item carried 4 points, while three single-choice items were assigned 3 points each. Items in all other dimensions were valued at 1 point apiece, yielding a maximum total score of 50. Due to skip patterns in some multiple-choice questions, the number of items answered varied among respondents, resulting in different possible total scores for each respondent. Adequate OHL was defined as achieving ≥80% of the total possible score. The same criterion was applied to each dimension. The overall OHL level was determined as the proportion of participants who met the criterion for adequate OHL, and the same method was used to calculate dimension-specific OHL levels.

#### Independent variables

Enterprise characteristics included scale, ownership and industry. Scale was classified based on the total number of employees in accordance with the official standards of the National Bureau of Statistics of China (23). Specifically, enterprises with 1,000 or more employees were defined as large enterprises, and those with 300–999 employees were classified as medium enterprises. In line with the statistical standards, enterprises with 20–299 employees are considered small enterprises, and those with fewer than 20 employees are considered micro enterprises. However, in this study, due to the relatively small number of micro enterprises (*n* = 116), small and micro enterprises were combined into a single category (enterprises with fewer than 300 employees) for the purposes of analysis. Ownership was classified into three categories: state-owned enterprises, private enterprises, and foreign/Hong Kong-Macao-Taiwan (HKMT)-funded enterprises, based on the registration types specified by the Administration for Industry and Commerce (24). Industry was divided into three categories according to the main business activities of the enterprises: environmental sanitation, transportation, express and food delivery.

### Covariates

Potential confounders included sex (male and female), age (<30, 30–39, 40–49, and ≥50 years), ethnicity (Han, others), marital status (married, unmarried, others), hukou type (agricultural, Non-agricultural), education level (primary education or below, lower secondary education, upper secondary education, tertiary education), monthly income (<3,000, 3,000–4,999, 5,000–6,999, and ≥7,000 CNY), and working years (<2, 2–<4, 4–<6, and ≥6 years).

### Statistical analysis

Descriptive statistics were used to summarize all variables, with categorical variables expressed as frequencies and percentages. The distribution of overall OHL and its dimensions across enterprise characteristics was summarized. Chi-square tests were used to examine differences in OHL and its dimensions among enterprise characteristics. Logistic regression models were employed to assess associations between enterprise characteristics and OHL, as well as its four dimensions, with both crude and adjusted odds ratios (ORs) and 95% confidence intervals (CIs) reported. Adjusted models included sex, age, working years, ethnic group, marital status, hukou type, education level, monthly income, and other enterprise characteristics. Stratified analyses by monthly income and interaction analyses between enterprise characteristics and monthly income were also conducted. All analyses were performed using R software (version 4.4.2), with a two-sided *p*-value <0.05 considered statistically significant.

## Results

### Participant characteristics

A total of 2,640 participants were included in this study. Males predominated (70.8%), with most participants aged between 30–39 years (29.9%) and 40–49 years (29.1%). Most participants worked in small and micro enterprises (57.4%), under private ownership (53.7%), and were equally distributed across three industries: environmental sanitation, transportation, and express and food delivery services (33.3% each). The demographic and occupational characteristics of participants are detailed in Table 1.

**Table 1:** The demographic and occupational characteristics of participants.

Characteristics		*n* (%)
Sample size		2,640
Sex	Male	1870 (70.8)
	Female	770 (29.2)
Age (years)	<30	514 (19.5)
	30–39	790 (29.9)
	40–49	767 (29.1)
	≥50	569 (21.6)
Working years	<2	920 (34.8)
	2–<4	593 (22.5)
	4–<6	418 (15.8)
	≥6	709 (26.9)
Ethnic group	Han	2,538 (96.1)
	Minority	102 (3.9)
Marital status	Unmarried	550 (20.8)
	Married	1997 (75.6)
	Others	93 (3.5)
Hukou type	Agricultural	1,596 (60.5)
	Non-agricultural	1,044 (39.5)
Education level	Primary education or below	300 (11.4)
	Lower secondary education	853 (32.3)
	Upper secondary education	853 (32.3)
	Tertiary education	634 (24.0)
Monthly income (CNY)	<3,000	396 (15.0)
	3,000–4,999	979 (37.1)
	5,000–6,999	713 (27.0)
	≥7,000	552 (20.9)
Scale	Large	738 (28.0)
	Medium	387 (14.7)
	Small and Micro	1,515 (57.4)
Ownership	State-owned	754 (28.6)
	Private	1,417 (53.7)
	Foreign/HKMT	469 (17.8)
Industry	Environmental Sanitation	880 (33.3)
	Transportation	880 (33.3)
	Express and Food Delivery	880 (33.3)

HKMT, Hong Kong, Macao, and Taiwan.

### OHL levels by enterprise characteristics

The overall OHL level among essential service workers was 52.8%, with the knowledge of occupational health protection dimension showing the highest level (77.6%) and skills of occupational health protection dimension showing the lowest (44.5%). As presented in Table 2, analysis across industries revealed no significant differences in overall OHL levels (*χ*^2^ = 0.149, *p* = 0.928), with levels of 53.3, 52.4, and 52.7% observed in environmental sanitation, transportation, and express and food delivery industries, respectively. However, significant differences were observed in the healthy work patterns and behaviors dimension (*p* < 0.001), where environmental sanitation workers having the highest level (64.2%) and express and food delivery workers the lowest (53.6%). In terms of enterprise characteristics, small and micro enterprises showed significantly higher OHL levels (56.9%) compared to large enterprises (47.0%) (*χ*^2^ = 23.981, *p* < 0.001). Furthermore, significant variations were found across ownership types (*χ*^2^ = 46.943, *p* < 0.001), with foreign/HKMT-funded enterprises showing the highest OHL level (60.1%), followed by private enterprises (55.8%), while state-owned enterprises demonstrated the lowest level (42.6%).

**Table 2:** Distribution of occupational health literacy and its dimensions by enterprise characteristics.

Enterprise characteristics	Occupational health literacy		Knowledge of occupational health laws		Knowledge of occupational health protection		Skills of occupational health protection		Healthy work patterns and behaviors	
	Adequate *n* (%)	Inadequate *n* (%)	Adequate *n* (%)	Inadequate *n* (%)	Adequate *n* (%)	Inadequate *n* (%)	Adequate *n* (%)	Inadequate *n* (%)	Adequate *n* (%)	Inadequate *n* (%)
Total	1,394 (52.8)	1,246 (47.2)	1,338 (50.7)	1,302 (49.3)	2048 (77.6)	592 (22.4)	1,174 (44.5)	1,466 (55.5)	1,550 (58.7)	1,090 (41.3)
Scale										
Large	347 (47.0)	391 (53.0)	338 (45.8)	400 (54.2)	582 (78.9)	156 (21.1)	266 (36.0)	472 (64.0)	403 (54.6)	335 (45.4)
Medium	185 (47.8)	202 (52.2)	182 (47.0)	205 (53.0)	288 (74.4)	99 (25.6)	166 (42.9)	221 (57.1)	206 (53.2)	181 (46.8)
Small and Micro	862 (56.9)	653 (43.1)	818 (54.0)	697 (46.0)	1,178 (77.8)	337 (22.2)	742 (49.0)	773 (51.0)	941 (62.1)	574 (37.9)
*χ* ^2^	23.981		15.752		2.947		34.072		17.153	
*P*	<0.001		<0.001		0.229		<0.001		<0.001	
Ownership										
State-owned	321 (42.6)	433 (57.4)	318 (42.2)	436 (57.8)	562 (74.5)	192 (25.5)	263 (34.9)	491 (65.1)	384 (50.9)	370 (49.1)
Private	791 (55.8)	626 (44.2)	746 (52.6)	671 (47.4)	1,124 (79.3)	293 (20.7)	656 (46.3)	761 (53.7)	878 (62.0)	539 (38.0)
Foreign/HKMT	282 (60.1)	187 (39.9)	274 (58.4)	195 (41.6)	362 (77.2)	107 (22.8)	255 (54.4)	214 (45.6)	288 (61.4)	181 (38.6)
*χ*2	46.943		35.259		6.352		48.607		26.424	
*P*	<0.001		<0.001		0.038		<0.001		<0.001	
Industry										
Environmental sanitation	469 (53.3)	411 (46.7)	469 (53.3)	411 (46.7)	667 (75.8)	213 (24.2)	372 (42.3)	508 (57.7)	565 (64.2)	315 (35.8)
Transportation	461 (52.4)	419 (47.6)	434 (49.3)	446 (50.7)	700 (79.5)	180 (20.5)	407 (46.2)	473 (53.8)	513 (58.3)	367 (41.7)
Express and food delivery	464 (52.7)	416 (47.3)	435 (49.4)	445 (50.6)	681 (77.4)	199 (22.6)	395 (44.9)	485 (55.1)	472 (53.6)	408 (46.4)
*χ*2	0.149		3.610		3.584		2.911		20.367	
*P*	0.928		0.164		0.167		0.233		<0.001	

HKMT, Hong Kong, Macao, and Taiwan.

### Associations between enterprise characteristics and OHL

Logistic regression analyses revealed significant associations between enterprise characteristics and OHL dimensions (Table 3). In terms of enterprise scale, small and micro enterprises showed positive associations compared to large enterprises, with crude OR of 1.49 (95%CI:1.25–1.78, *p* < 0.05) for overall OHL, which remained significant after adjusting for demographic and other enterprise characteristics (adjusted OR = 1.42, 95%CI: 1.17–1.72, *p* < 0.05). This pattern was particularly in Skills of Occupational Health Protection (adjusted OR = 1.56, 95%CI: 1.28–1.90, *p* < 0.05). Regarding ownership, private enterprises demonstrated positive association with overall OHL compared to state-owned enterprises (adjusted OR = 1.28, 95%CI: 1.05–1.55, *p* < 0.05), while foreign/HKMT enterprises showed negative association in Healthy Work Patterns and Behaviors dimension (adjusted OR = 0.83, 95%CI: 0.72–0.95, *p* < 0.05). For industries, compared to environmental sanitation industry, express and food delivery industry showed significant negative associations in both Healthy Work Patterns and Behaviors dimension (adjusted OR = 0.53, 95%CI: 0.41–0.68, *p* < 0.05) and overall OHL (adjusted OR = 0.64, 95%CI: 0.49–0.82, *p* < 0.05). Transportation sector also demonstrated negative association in Healthy Work Patterns and Behaviors dimension (adjusted OR = 0.68, 95%CI: 0.54–0.86, *p* < 0.05).

**Table 3:** Association between enterprise characteristics and different dimensions of occupational health literacy.

Enterprise characteristics	Occupational health literacy		Knowledge of occupational health laws		Knowledge of occupational health protection		Skills of occupational health protection		Healthy work patterns and behaviors	
	Crude OR (95%CI)	Adjusted OR (95%CI)	Crude OR (95%CI)	Adjusted OR (95%CI)	Crude OR (95%CI)	Adjusted OR (95%CI)	Crude OR (95%CI)	Adjusted OR (95%CI)	Crude OR (95%CI)	Adjusted OR (95%CI)
Scale										
Large	1.00 (Ref)	1.00 (Ref)	1.00 (Ref)	1.00 (Ref)	1.00 (Ref)	1.00 (Ref)	1.00 (Ref)	1.00 (Ref)	1.00 (Ref)	1.00 (Ref)
Medium	1.03 (0.81–1.32)	1.06 (0.81–1.38)	1.05 (0.82–1.34)	1.03 (0.79–1.35)	0.78 (0.58–1.04)	0.84 (0.62–1.14)	1.33 (1.04–1.71)*	1.46 (1.12–1.91)*	0.95 (0.74–1.21)	0.93 (0.72–1.21)
Small and micro	1.49 (1.25–1.78)*	1.42 (1.17–1.72)*	1.39 (1.16–1.66)*	1.34 (1.10–1.63)*	0.94 (0.75–1.16)	1.03 (0.82–1.29)	1.70 (1.42–2.04)*	1.56 (1.28–1.90)*	1.36 (1.14–1.63)*	1.44 (1.18–1.75)*
Ownership										
State-owned	1.00 (Ref)	1.00 (Ref)	1.00 (Ref)	1.00 (Ref)	1.00 (Ref)	1.00 (Ref)	1.00 (Ref)	1.00 (Ref)	1.00 (Ref)	1.00 (Ref)
Private	1.65 (1.40–1.95)*	1.28 (1.05–1.55)*	1.59 (1.35–1.88)*	1.21 (1.00–1.48)	1.11 (0.92–1.34)	1.13 (0.90–1.41)	1.76 (1.49–2.08)*	1.35 (1.11–1.64)*	1.35 (1.15–1.60)*	1.17 (0.96–1.42)
Foreign/HKMT	0.86 (0.76–0.98)*	0.91 (0.79–1.05)	0.93 (0.82–1.05)	0.96 (0.83–1.10)	0.85 (0.73–0.99)*	0.89 (0.75–1.05)	0.94 (0.83–1.07)	1.00 (0.87–1.15)	0.82 (0.72–0.94)*	0.83 (0.72–0.95)
Industry										
Environmental sanitation	1.00 (Ref)	1.00 (Ref)	1.00 (Ref)	1.00 (Ref)	1.00 (Ref)	1.00 (Ref)	1.00 (Ref)	1.00 (Ref)	1.00 (Ref)	1.00 (Ref)
Transportation	0.96 (0.80–1.16)	0.80 (0.63–1.01)	0.85 (0.71–1.03)	0.80 (0.63–1.02)	1.24 (0.99–1.56)	0.88 (0.66–1.16)	1.18 (0.97–1.42)	1.00 (0.79–1.27)	0.78 (0.64–0.94)*	0.68 (0.54–0.86)*
Express and food delivery	0.98 (0.81–1.18)	0.64 (0.49–0.82)*	0.86 (0.71–1.03)	0.72 (0.56–0.94)*	1.09 (0.88–1.36)	0.66 (0.48–0.90)*	1.11 (0.92–1.34)	0.71 (0.55–0.92)*	0.64 (0.53–0.78)*	0.53 (0.41–0.68)*

*P < 0.05 was considered statistically significant; OR: odds ratio; 95%CI: 95% confidence interval; Crude: no adjustment; Adjusted: adjusted for sex, age, working years, ethnic group, marital status, hukou type, education level, monthly income and other enterprise characteristics; HKMT, Hong Kong, Macao, and Taiwan.

### Stratified analyses

**Figure 2:**
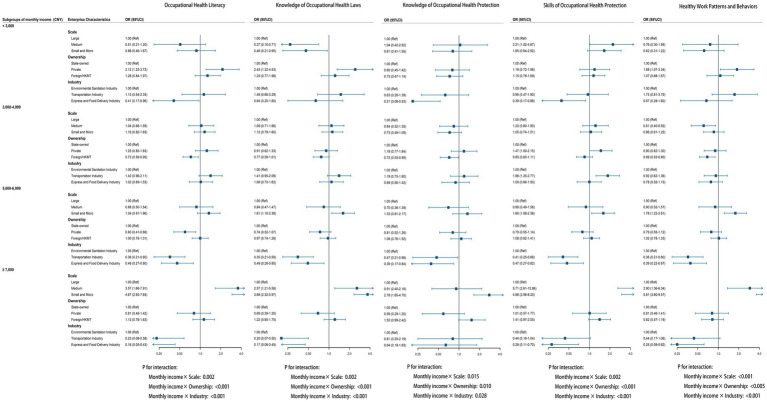
Associations between enterprise characteristics and occupational health dimensions stratified by monthly income. OR: odds ratio; 95%CI: 95% confidence interval; Adjusted for sex, age, working years, ethnic group, marital status, hukou type, education level, monthly income and other enterprise characteristics; HKMT: Hong Kong, Macao, and Taiwan; *P_for interaction_* were calculated between monthly income and enterprise scale, ownership, and industry, respectively.

Stratified analyses revealed significant interactions between monthly income levels and enterprise characteristics in relation to OHL and its dimensions (all *P*_for interaction_<0.05, Figure 2). The association between small/micro enterprises and OHL was markedly stronger in the high-income subgroup (≥7,000 CNY) with an adjusted OR of 4.67 (95% CI: 2.92–7.65) compared to other income subgroups. The relationship between ownership and OHL, particularly knowledge of occupational health laws, was significantly positive only in private enterprises within the low-income subgroup (<3,000 CNY). The negative association between industry and OHL was only statistically significant only in higher income subgroups, suggesting that industry-specific factors predominantly influence OHL among higher-income workers.

## Discussion

This surveillance study provides important insights into OHL among essential service workers in Guangdong Province, China. Our findings revealed a relatively low overall OHL level of 52.8%, significant associations between enterprise characteristics (scale, ownership, and industry) and OHL levels, as well as notable moderating effects of workers’ monthly income on these relationships. These results highlight the complex interplay between enterprise characteristics and monthly income in shaping OHL among essential service workers, emphasizing the importance of considering both enterprise factors and income levels in OHL surveillance and the development of targeted interventions.

Inadequate health literacy is a universal problem both in the general and working populations, and it may be restricting the workers understanding of occupational health and safety training (25). The overall OHL level of 52.8% among essential service workers in this study is higher than the tertiary industry national average (48.9%), but lower than that of secondary industry workers (56.5%) (26). Unlike workers in more regulated industries, essential service workers often have limited access to occupational health resources, training opportunities, and organizational support systems, which may further exacerbate their vulnerability to occupational risks. While unadjusted analysis showed similar OHL levels across environmental sanitation (53.3%), transportation (52.4%), and express and food delivery industries (52.7%), multivariate analysis revealed significant differences after controlling for confounding factors. Environmental sanitation workers demonstrated the highest level in healthy work patterns and behaviors (64.2%) and knowledge of occupational health protection (75.8%). This strong performance could be attributed to the standardization of sanitation work and implementation of comprehensive occupational health management systems in recent years. Our findings show similar patterns with previous research, where approximately 70.7% of sanitation workers showed moderate levels of knowledge in preventing occupational health risks, and 70.1% reported regular use of personal protective equipment during work (27). These results suggest that sanitation workers generally maintain relatively good levels of occupational health knowledge and protective behaviors. Transportation workers showed moderate levels of occupational health literacy (52.4%) and knowledge of occupational health protection (79.5%), yet their healthy work behaviors (58.3%) were lower than environmental sanitation workers. This gap between knowledge and behavior deserves attention. Transportation workers typically receive safety training and daily health education, which may explain their moderate knowledge levels. However, a study has shown that these workers, particularly online ride-hailing drivers, are characterized by working long hours with few breaks (28). Such intensive work patterns may contribute to fatigue and reduced adherence to healthy work practices despite having adequate health knowledge. Notably, express and food delivery workers demonstrated significantly lower OHL levels in the adjusted models. This finding aligns with previous research highlighting the precarious working conditions in the rapidly expanding gig economy (29). Unlike workers in more traditional sectors, express and food delivery personnel often operate as independent contractors rather than formal employees, which may limit their access to systematic occupational health training and organizational support systems (30). Additionally, express and food delivery workers usually operate under intense time pressures, and irregular work schedules, which may limit their opportunities to engage in health-promoting activities and access occupational health resources (31, 32). The relatively lower OHL compared to secondary industry may be attributable to inadequate health training and weaker protection systems, highlighting the need for targeted interventions in these essential service sectors.

Further analysis revealed that enterprise scale was also significantly associated with OHL. Specifically, our study found that workers from small and micro enterprises had higher OHL levels compared to those from large enterprises, which contrasts with previous research suggesting that smaller enterprises usually have limited resources for occupational health services and training (19). One possible explanation for this discrepancy might be the implementation of the “Occupational Health Support Initiative for Small, Medium and Micro-sized Enterprises” in recent years, which has promoted the standardization of occupational health management and increased workers’ awareness of occupational health protection. This initiative may have significantly improved the accessibility and effectiveness of occupational health education and training within smaller enterprises (33). Moreover, smaller enterprises might have closer interpersonal relationships and communication channels between management and employees, facilitating more effective dissemination of occupational health information and greater responsiveness to health concerns (34). This trend was particularly evident among workers with a monthly income ≥7,000 CNY, possibly because higher-income employees tend to have greater health awareness and are more likely to benefit from occupational health initiatives implemented in small and micro enterprises (35).

Our results regarding enterprise ownership and OHL present a contrast to existing literature. We found that workers in private enterprises had significantly higher overall OHL compared to those in state-owned enterprises, whereas previous studies typically reported better occupational health practices and resources in state-owned enterprises due to their greater regulatory oversight and resource availability (26). This unexpected finding may reflect evolving management practices in China’s private sector, where competitive market pressures increasingly incentivize enterprises to prioritize employee health as a critical factor for productivity and corporate reputation (36, 37). Interestingly, our stratified analysis indicated that this positive association between private ownership and OHL was particularly pronounced among low-income workers. Private enterprises might adopt more targeted and flexible health promotion strategies tailored to lower-income workers, who are often at greater occupational risk and have limited access to external health resources.

A key strength of this study is its focus on essential service workers—a population often overlooked in occupational health research—as well as its comprehensive examination of the influence of enterprise characteristics on occupational health literacy. Several limitations should be acknowledged. First, due to the cross-sectional nature of the data, the temporal sequence between enterprise characteristics and OHL cannot be established, and thus, causality cannot be inferred from the observed associations. Second, although participants were recruited from several key industries, the sample size was relatively small compared to the total population of essential service workers in Guangdong Province. However, using G*Power 3.1.9.7, a post-hoc power analysis revealed that our sample size of 2,640 participants provided adequate statistical power (85.05%) to detect an odds ratio of 1.28 (*α* = 0.05, two-tailed), suggesting our study was sufficiently powered to detect the hypothesized effects. Nevertheless, the relatively small sample size may still limit the representativeness of our findings, thereby restricting the generalizability of our conclusions to all essential service workers in the region. Finally, while our study examined several key enterprise characteristics, other potentially important organizational factors—such as leadership commitment to health, workplace social support, and specific occupational health management practices—were not assessed. These factors may mediate or moderate the relationships between enterprise characteristics and OHL and warrant investigation in future studies. To address these limitations, future research should consider recruiting larger and more representative samples across different geographic regions and occupational groups, thereby enhancing the external validity of the findings. Moreover, adopting longitudinal study designs would allow for a better understanding of the causal relationships between enterprise characteristics and OHL over time. In addition, mixed-methods approaches—combining quantitative surveys with in-depth qualitative interviews—could provide deeper insights into workers’ lived experiences and the organizational cultures that facilitate or hinder improvements in occupational health literacy (38). Furthermore, intervention trials are warranted to evaluate the effectiveness of innovative strategies, such as mobile-based learning, gamification, or AI-driven personalized health promotion, in diverse enterprise settings (39–41). Such studies would not only help identify best practices for promoting OHL among essential service workers, but also inform the development of scalable and sustainable interventions tailored to the unique needs of different worker populations.

Importantly, our findings underscore the need for differentiated policy approaches across various enterprise types to enhance occupational health literacy. For the express and food delivery sector, where OHL levels are significantly lower, policymakers should prioritize the establishment of mandatory occupational health training programs and standardized health protection protocols, particularly focusing on platform companies’ responsibilities toward gig workers (42, 43). The successful standardized occupational health management systems in the environmental sanitation sector could serve as a model for other industries, while the transportation sector requires specific policies to bridge the gap between knowledge and behavioral practices, such as strengthening work hours regulation. The demonstrated success of the “Occupational Health Support Initiative for Small, Medium and Micro-sized Enterprises” suggests the value of continuing and expanding such targeted support programs, while large enterprises should be encouraged to adopt more personalized and interactive approaches to health education (44). Given the positive outcomes observed in private enterprises, particularly among lower-income workers, policymakers should promote the sharing of best practices across ownership types, encouraging state-owned enterprises to incorporate more flexible and targeted approaches to health promotion. These targeted policy interventions, tailored to specific enterprise characteristics, could significantly improve occupational health literacy and ultimately enhance worker well-being across essential service sectors.

## Conclusion

This surveillance study identified that enterprise characteristics are significantly associated with OHL among essential service workers in Guangdong Province, China. Employment in small/micro enterprises and private enterprises was associated with higher OHL levels, while working in the express and food delivery industry was associated with lower OHL levels. These findings highlight the need to consider enterprise characteristics in occupational health surveillance and when developing targeted interventions to improve OHL. Future longitudinal studies are needed to clarify the causal mechanisms of these associations and to evaluate the effectiveness of enterprise-targeted OHL interventions.

## Data Availability

The raw data supporting the conclusions of this article will be made available by the authors, without undue reservation.

## Footnotes

^1^ https://zyjksy.niohpinfo.cn/
